# PK20, a new opioid-neurotensin hybrid peptide that exhibits central and peripheral antinociceptive effects

**DOI:** 10.1186/1744-8069-6-86

**Published:** 2010-12-06

**Authors:** Patrycja Kleczkowska, Piotr Kosson, Steven Ballet, Isabelle Van den Eynde, Yuko Tsuda, Dirk Tourwé, Andrzej W Lipkowski

**Affiliations:** 1 Mossakowski Medical Research Centre, Polish Academy of Sciences, Pawinskiego Street 5, 02106 Warsaw, Poland; 2 Department of Organic Chemistry, Vrije Universiteit Brussel, Brussels, Belgium; 3 Faculty of Pharmaceutical Sciences, Kobe Gakuin University, Kobe, Japan

## Abstract

**Background:**

The clinical treatment of various types of pain relies upon the use of opioid analgesics. However most of them produce, in addition to the analgesic effect, several side effects such as the development of dependence and addiction as well as sedation, dysphoria, and constipation. One solution to these problems are chimeric compounds in which the opioid pharmacophore is hybridized with another type of compound to incease antinociceptive effects. Neurotensin-induced antinociception is not mediated through the opioid system. Therefore, hybridizing neurotensin with opioid elements may result in a potent synergistic antinociceptor.

**Results:**

Using the known structure-activity relationships of neurotensin we have synthesized a new chimeric opioid-neurotensin compound PK20 which is characterized by a very strong antinociceptive potency. The observation that the opioid antagonist naltrexone did not completely reverse the antinociceptive effect, indicates the partial involvement of the nonopioid component in PK20 in the produced analgesia.

**Conclusions:**

The opioid-neurotensin hybrid analogue PK20, in which opioid and neurotensin pharmacophores overlap partially, expresses high antinociceptive tail-flick effects after central as well as peripheral applications.

## Background

The opioids system is the major endogenous pathway that modulates pain signal transmission and perception. Therefore most pain medicines, available for the treatment of severe pain, express affinity for the opioid receptors. The search for selective opioid compounds is still a main avenue in the development of new analgesics. However, pain is mediated by various complementary and/or alternative pathways that participate in the creation of a final level of pain perception. Therefore, we have proposed a new, multitarget approach in searching for new analgesics [1]. According to this concept, the new type of analgesics should interact with a broad spectrum of pathways involved in pain transmission and modulation. The use of a peptidomimetic in the chemical structure of the drug allows to modulate the permeability of the active substance and, in consequence, creates "site specificity of action". This concept has been positively proven with the creation of multitarget molecules interacting with broad spectrum of opioid receptors [2] or opioid and NK_1_-, [3,4] CCK-,[5] or neurotensin receptors [6].

The tridecapeptide neurotensin, (NT, *p*-Glu^1^-Leu^2^-Tyr^3^-Glu^4^-Asn^5^-Lys^6^-Pro^7^-Arg^8^-Arg^9^-Pro^10^-Tyr^11^-Ile^12^-Leu^13^) [7-10] exerts antinociceptive activity, and is therefore considered as a pain modulating factor [11]. Microinjection experiments have provided evidence that NT can modulate pain transmission in several brain regions and pathways that are involved in the central integration of pain responses, including the central amygdale, the hypothalamic medial preoptic nucleus (MPO), certain thalamic nuclei, the periaqueductal gray (PAG), and the rostroventral medulla (RVM) [11,12]. Interestingly, neurotensin has bipolar (facilitatory and inhibitory) effects on pain modulation, which depend on the injected doses. Facilitation predominates at low (picomolar) doses of NT injected into the RVM, whereas higher doses (nanomolar) produce antinociception [13]. Until now, the NTS1 and NTS2 receptor subtypes, which belong to the class of G protein-coupled receptors, appear to be required for different aspects of neurotensin-induced analgesia [11,14,15].

When administered intracerebroventricularly (i.c.v.), NT induces a strong naloxone-insensitive analgesic effect [16-18], suggesting that NT operates independently of opioidergic transmission.

Structure-activity relationship studies with a number of neurotensin analogs and partial sequences have established that the C-terminal hexapeptide of NT contains all the structural requirements for receptor binding and activation.

**Figure 1:**
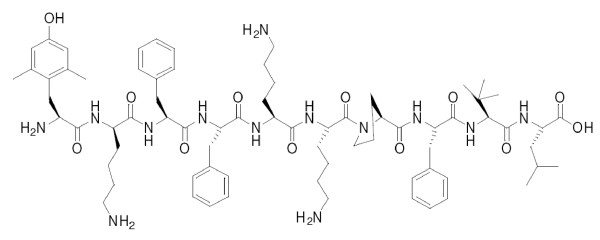
**Structure of PK20, a hybrid opioid-neurotensin decapeptide (H-Dmt-D-Lys-Phe-Phe-Lys-Lys-Pro-Phe-Tle-Leu-OH)**.

Moreover, the introduction of altered amino acids, relative to the native sequence, into the peptide, can improve the metabolic stability [19-21] and have a crucial influence on its function and activity [22,23]. In contrast to NT, the *N*-terminal fragment is crucial for the interaction of opioid peptides with all opioid receptors. Sequence analysis of the active fragment of both peptides resulted in the development of new analogues, in which the active opioid and neurotensin fragments partially overlap. Therefore, the final compounds should express independent affinities to opioid and nociceptin receptors [24]. The most potent compound of this series, PK20, has several modifications of parent fragments of endogenous peptides. The C-terminal fragment was modified by substitution of Arg^8 ^and Arg^9 ^by lysine residues, substitutions that are known to leave potency unchanged [22], and an additional substitution of Ile^12 ^by Tle (tert-leucine) that should improve the metabolic stability [25]. The endomorphin-2 pharmacophore, on the other hand, has been used as the parent opioid sequence. However, to improve its enzymatic stability and receptor affinity the *N*-terminal Tyr^1 ^has been replaced by 2,6, dimethyl-tyrosine (Dmt) and Pro^2 ^has been replaced by D-lysine. The final structure of PK20 is presented in Figure 1. This paper describes the antinociceptive properties of PK20 in the acute tail flick test after central (intrathecal, i.t.) application in rats and after peripheral (intravenous, i.v.) application in mice.

## Methods

### Measurement of antinociception by intrathecally administered hybrid PK20 into rats

PK20, the new opioid-neurotensin hybrid peptide was injected intrathecally into male Wistar rats (weighing 225-250 g) by way of implanted cannulae. Animals were housed separately and given full access to food and water. The technique of intrathecal drug administration, originally described by Yaksh & Rudy [26], was used to test the spinal action of the investigated opioid-neurotensin hybrid peptide, PK20. The analgesic activity of PK20 was evaluated by using the tail-flick test (Model 33 Tail Flick Analgesia Meter, USA), in which the role of the nociceptor agent was fulfilled by a light beam [27]. The measurement parameters (beam temperature, the time of rat's tail exposition on the irrigation of laser beam) were set properly in order to avoid the burn of tails.

The effect of action of PK20 was measured at different doses per rat and within a time frame of 120 minutes (at 5, 15, 30, 60 and 120 minutes after injection). For each time period three measurements were carried out.

Control responses for each rat in the tail-flick test, and for each mouse in hot water tail-flick test, were determined before the injection. Following an intrathecal injection of saline, morphine, and naltrexone, the response to PK20 was determined. Regarding naltrexone, PK20 was i.t. administered after 10 min interval after the injection of the opioid blocker.

The tail-flick test was scored as the percent of maximal possible effect (% MPE) and calculated using the following equation:

% MPE=(pdr−br)/(co−br)×100%

where pdr - post drug response, br - baseline response, co - cut-off value. The cut-off was 7 s for all experiments (n equals 6).

### Measurement of antinociception by intravenously administered hybrid PK20 into mice

Measurements of antinociception were also carried out by intravenous administration of PK20 into SWISS WEBSTER male white mice (weighing 35 - 40 g) and using the hot water tail-flick test. The hybrid compound was injected at a dose of 10 and 4 mg/kg. The effect of PK20 was measured within a period of 120 minutes (at 5, 15, 30, 60 and 120 minutes after injection). For each time period three measurements were carried out.

Hot water tail-flick measurements were taken in water warmed to a temperature of 55.5°C ± 1°C. Latency was measured as the time that the mice needed to remove its tail after it was placed into hot water.

The hot water tail-flick test was scored as % MPE, as was mentioned above. However in this case the applied cut-off was 10 s.

### Statistical analysis

All reported data represent the mean % MPE ± SEM. Significant difference at individual time points between two groups was determined by Student's T-test with use of Statistica^® ^7.1 software (StatSoft, Tulsa, USA) with *p < 0.05 and **p < 0.001 being considered significant.

## Results

### Antinociceptive effects of intrathecally administered PK20 hybrid peptide in tail-flick test

**Figure 2:**
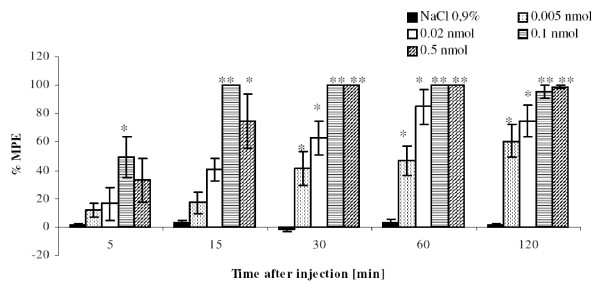
**Effect of increasing doses of hybrid peptide PK20 on analgesia in the rat tail-flick test**. The test was performed within 2 h after the i.t. injection of the compound. % MPE ± SEM of six animals per group. *p < 0.05; **p < 0.001 as compared to saline controls.

**Figure 3:**
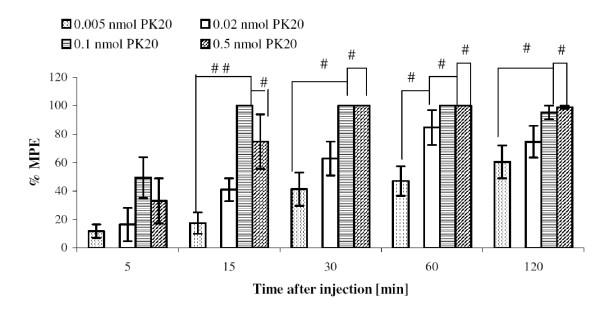
**Comparison of 0.005 nmol PK20 to other doses of peptide used in the experiment**. PK20 was injected intrathecally at doses of 0.02, 0.1 and 0.5 nmol, respectively; % MPE ± SEM of six animals per group. ^#^p < 0.05; ^# #^p < 0.001 versus 0.02, 0.1 and 0.5 nmol PK20/rat.

**Figure 4:**
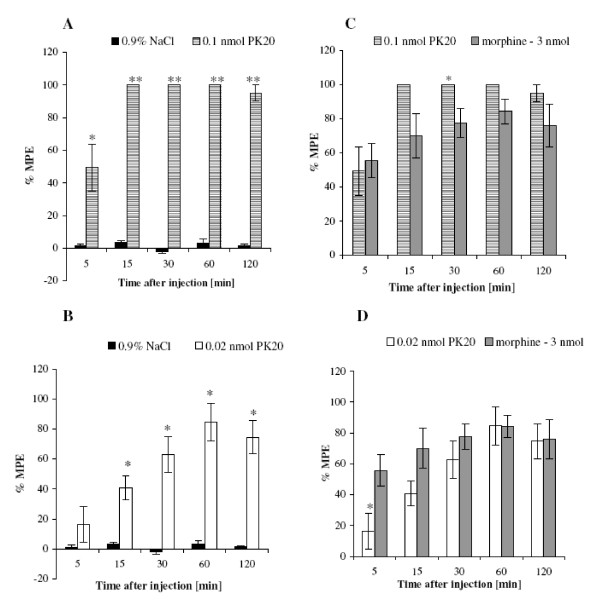
**The effect of different intrathecal doses of PK20 (0.02, 0.1 nmol) vs. morphine (3 nmol) and with 0.9% NaCl as control**. Each column represents mean ± SEM of 6 animals. *p < 0.05; **p < 0.001 versus saline-injected (A, B); *p < 0.05; **p < 0.001 versus morphine.

In this experiment, increasing doses of PK20 (0.5-0.005 nmol/rat) were administered and its analgesic effect was evaluated within a period of 2 hours by means of the tail-flick test. In Figure 2 a dose-dependent antinociceptive effect of PK20 is presented. It is shown, that by increasing doses of the administered peptide, an increasing effect of antinociception was observed only in a lower doses, up to 0.1 nmol. The higher dose of 0.5 nmol did not produce a greater effect than 0.1 nmol. Animals injected with the investigated peptide showed a statistically significant increase of the % MPE value compared to the control group. Interestingly, the analgesic effect induced by PK20 at a dose of 0.1 nmol/rat noted at 5 min after injection was larger than for PK20 at a dose 0.5 nmol per rat (at the same time point), however there was no significant difference observed (P = 0.46676, t-experiment = 0.764071). Still, in further measurements, starting from 30 minutes after peptide injection, values of % MPE were the same as for its lower doses. In Figure 3 the comparison of each dose to PK20 at 0.005 nmol/rat, is shown. We have also compared two doses of PK20 (0.1 and 0.02 nmol/rat, respectively) with morphine (3 nmol/rat) as a control group. As is shown in Figure 4C and 4D, the analgesic strength of the peptide in both doses is almost similar to this observed for i.t. injected morphine at a dose of 3 nmol per rat. For a dose of 0.1 nmol of the opioid-neurotensin hybrid we observed a much greater analgesic activity than for morphine's higher concentration. Interestingly, at 60 min after administration of a lower dose of PK20 (0.02 nmol/rat), the same value of % MPE for both morphine and PK20, is obtained.

To evaluate the possible analgesic effect of the neurotensin part of PK20, a μ-opioid receptor blocker, naltrexone, which antagonizes the antinociceptive effect, induced by the opioid subunit in PK20, was used.

**Figure 5:**
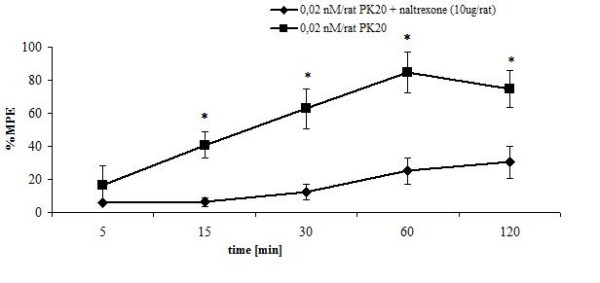
**Time-course of the antinociceptive effect of peptide PK20 alone and PK20 after a naltrexone injection**. A. analgesic effect of chimeric compound PK20 injected intrathecally at a dose of 0.02 nmol/rat; B. analgesic effect of PK20 (0.02 nmol), injected 10 min after a naltrexone (10 μg/rat) injection. % MPE ± SEM of six animals per group. *p < 0.05; **p < 0.001 as compared to rats treated with PK20 (0.02 nmol/rat) alone.

In Figure 5 a comparison is presented between the effect of PK20 at a dose of 0.02 nmol/rat (at which the analgesic effect is observed) and the analgesic effect of PK20 (0.02 nmol/rat) administered 10 minutes after a naltrexone injection (10 μg/rat).

It was observed that, although naltrexone significantly reduced the analgesic effect induced by PK20, the growing profile of PK20's antinociceptive action is still preserved.

### Effects of PK20 hybrid peptide in hot water tail-flick test after intravenous application in mice

To evaluate PK20's ability to act after peripheral administration, and thus to cross the blood-brain barrier (BBB), we examined the analgesic effect induced by intravenous application of the peptide at two doses of 4 and 10 mg/kg, respectively.

**Figure 6:**
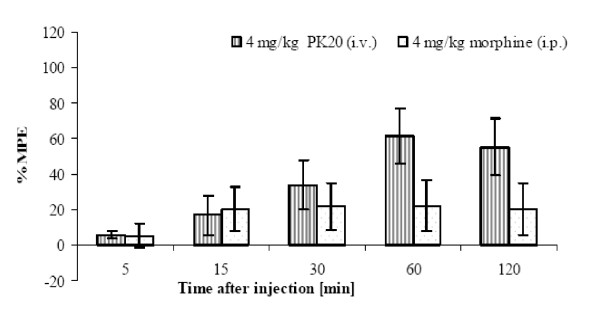
**Time-course of the antinociceptive effect of intravenously administered PK20 (4 mg/kg) vs. intraperitoneally injected morphine (4 mg/kg)**. Each column represents the mean value ± SEM of 6 - 8 animals. *p < 0.05; **p < 0.001, significantly different from morphine-injected animals.

**Figure 7:**
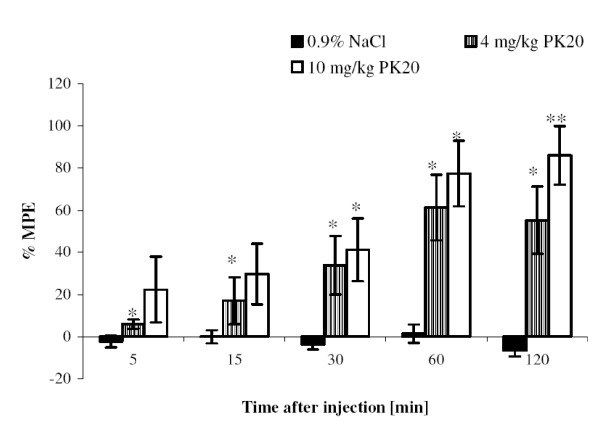
**Time-course of the antinociceptive effect of PK20 after i.v. administration (4 mg/kg and 10 mg/kg, respectively) vs. intravenously administered of 0.9% NaCl solution**. Each column represents mean ± SEM of 6 - 8 animals. *p < 0.05; **p < 0.001, significantly different from saline-injected mice.

The obtained results clearly indicate that the opioid-neurotensin hybrid peptide PK20 can exert an analgesic effect after intravenous injection. Additionally, a growing time-dependent effect is still observed. When comparing PK20 to morphine, injected into mice at the same dose (Figure 6), there is no significant difference between those two drugs. However, In contrast to Figure 6 when compared to saline, both administered doses of PK20 demonstrate significant differences (*p < 0.05), which is shown in Figure 7.

## Discussion

Our *in vivo *studies have shown that PK20 treatment results in long-standing time-dependent antinociception when administered centrally as well as peripherally. This novel opioid-neurotensin hybrid peptide has a significantly intensified analgesic effect, when compared to saline and morphine. The improved analgesia mediated by this peptide suggests a possible plasma stability and implies a delayed enzymatic degradation (data not published). Intrathecal injection of PK20 at a dose of 0.02 nmol/rat exerts a similar analgesic action to that observed for morphine at 3 nmol/rat, indicating a very high antinociceptive potency of the investigated peptide. Interestingly, by increasing the concentration of the administered compound (range between 0.1-0.5 nmol/rat) the antinociceptive effect at 0.5 nmol/rat starts from a lower value than at 0.1 nmol/rat during the first 15 minutes after injection, which might be interpreted as a short delay in response to nociceptive stimuli.

Naltrexone, injected 10 min before the compound, effectively attenuated the antinociceptive action produced by the hybrid peptide. However, a slight and still time-dependent growing profile of analgesic effect of PK20 is still preserved. These findings indicate that naltrexone only partially inhibited the antinociceptive action of PK20, suggesting that also the neurotensin fragment is involved in analgesia. The analgesic response induced by PK20 is mediated not only through activation of the opioid pathway, but also through action at neurotensin receptors.

Our study also indicates the ability of PK20 to cross the highly selective blood-brain barrier, which was examined by its intravenous administration into mice (hot water tail-flick test). Since only a few neurotensin analogs were reported to cross the BBB, like a compound from the Eisai group [23] or NT66L and NT69L [19,28], PK20 seems to be a very interesting novel pain relieving drug.

Neurotensin as well as opioids exert analgesia. Opioids drugs block pain signals by interacting especially with mu-opioid receptors, whereas NT or its analogs act independently of the opioid pathways. Therefore, by combining these two elements, the antinociceptive effect might be obtained either by the opioid or neurotensin part alone, or by synergy of two interacting parts, thus acting more efficiently than in case of separate administration of each of them.

Having in mind the fact that chronic administration of opiates generally produce tolerance [29,30] and dependence, which are highly undesirable effects, the creation of novel compounds such as this hybrid may have an influence on the reduction of these side-effects and gives a hope to obtain new drugs with the ability to sufficiently relief pain states.

The comparative studies on tolerance development after multiple application of PK20 and morphine are in progress.

## Conclusions

The opioid-neurotensin hybrid analogue PK20, in which opioid and neurotensin pharmacophores partially overlap, expresses high antinociceptive tail-flick effects after central as well as peripheral application.

## Competing interests

The authors declare that they have no competing interests.

## Authors' contributions

All authors contribute equally in research and preparing the manuscript. All authors approved the final manuscript.
